# Very High Incidence of Type 1 Diabetes Among Children Aged Under 15 Years in Tlemcen, Northwest Algeria (2015-2018)

**DOI:** 10.4274/jcrpe.galenos.2020.2020.0073

**Published:** 2021-02-26

**Authors:** Sarra Khater, Ammaria Aouar, Nawel Bensmain, Salih Bendedouche, Nafissa Chabni, Houari Hamdaoui, Abdellatif Moussouni, Zakarya Moqaddem

**Affiliations:** 1 Abou Beker Belkaid University, Valorisation of Human Actions for the Protection of the Environment and Application in Public Health Laboratory, Tlemcen, Algeria; 2 Abou Beker Belkaid University, Statistics and Random Models Laboratory, Tlemcen, Algeria; 3 Abou Beker Belkaid University, Tlemcen University Hospital, Department of Pediatrics, Tlemcen, Algeria; 4 Abou Beker Belkaid University, Tlemcen University Hospital, Department of Epidemiology, Tlemcen, Algeria; 5 Abou Beker Belkaid University, Anthropology Laboratory, Tlemcen, Algeria

**Keywords:** Type 1 diabetes, children, incidence, Tlemcen, Northwest Algeria

## Abstract

**Objective:**

In Algeria, there is a lack of epidemiological data concerning childhood type 1 diabetes (T1D). The International Diabetes Federation estimated in 2019 that Algeria ranked 7th among countries with the highest prevalence of T1D. This study aimed to determine the incidence of T1D in children <15 years, living in Tlemcen in Northwest Algeria.

**Methods:**

A retrospective study using data from children (<15 years) who have been diagnosed with T1D in Tlemcen between 2015 and 2018, using the two-source capture–recapture method to estimate the completeness of ascertainment (%). Total average incidences, by sex, by onset age group, and by season of onset were calculated per 100,000 and per year.

**Results:**

During the study period, 437 new cases of T1D were registered, among them, 233 boys and 204 girls, with a sex ratio of 1.14. The average annual incidence rate of childhood T1D was 38.5/100,000 with a 95% confidence interval (CI): 35.20-41.79; boys: 40.51, 95% CI: 38.16-42.85; girls: 36.49, 95% CI: 34.17-38.80. Overall incidence rates in 2015, 2016, 2017 and 2018 were respectively 36.6 (95% CI: 33.72-39.48), 38.7 (95% CI: 35.43-41.97), 39.3 (95% CI: 35.97-42.62) and 39.5 (95% CI: 36.12-42.87)/100,000. Newly diagnosed children were more likely to present in winter and autumn. Ketoacidosis at diagnosis was diagnosed in 29.2%.

**Conclusion:**

The mean incidence of childhood T1D in Tlemcen was 38.5/100,000, this incidence is in the “extremely high” category of the World Health Organization DiaMond project classification of diabetes giving this region a very high risk.

**What is already known on this topic?**

Algeria ranked among the top 10 countries with highest number of children with type 1 diabetes (T1D) in 2019.

**What this study adds?**

This study is the first to report the incidence of T1D in children under 15 years in the region of Tlemcen in Northwest Algeria. The incidence of T1D in children under 15 years was 38.5/100,000 during 2015-2018 in the region of Tlemcen.

## Introduction

Type 1 diabetes (T1D) or insulin-dependent diabetes is the most common endocrine and metabolic disorder in children and represents 80-90% of diabetes in children and adolescents (1,2). Since 1950, the incidence of diabetes in children has increased substantially around the world. The World Health Organization (WHO) Multinational Project for Childhood Diabetes (WHO DiaMond Project) estimates an annual average increase at around 3% (3).

The incidence of T1D varies widely between countries, and even between regions of the same country; there is a geographical disparity in the epidemiological trends of childhood diabetes worldwide (4). The highest incidence rates were recorded in Finland, in Sweden and in Sardinia while East Asian and American Indians populations have the lowest rates (5). This geographic heterogeneity in incidence trends is due to factors such as variability in genetic predisposition and environmental factors for autoimmune destruction of beta-pancreatic cells (6,7). The role of environmental triggers in the development of childhood diabetes has been suggested because of the marked seasonal variation in the onset of childhood diabetes (8).

According to estimates in the 8^th^ Edition of the International Diabetes Federation Diabetes Atlas, Algeria ranks seventh in the world among countries with the highest estimated number of prevalent children aged under 15 years with T1D (n=20,100) (9). Algeria is also the country in the Middle East and North Africa (MENA) Region with the highest number of new cases (incidence) of T1D in this age group with 3,100 children in 2019 (10).

In Algeria there is a lack of available scientific data on the incidence and prevalence of T1D in children, with only three functional regional registries for T1D in children under 15 years of age (11). In Algiers (in north-central Algeria) the incidence of T1D among children under 15 rose from 22.3 per 100,000 in 2010 to 29.0 per 100,000 in 2015 (12). In Oran (northwest region) the incidence of T1D in children under 15 rose from 4.7 per 100,000 in the period 1979-1988 to 24.46 per 100,000 in the period 2010-2014 with an annual increase in incidence of 5.04 (13,14). In Constantine in the North East of Algeria the incidence of T1D in children under 15 increased from 9.57 per 100,000 between 1990-1994 to 17.44 per 100,000 in 2003 (15). However, in the region of Tlemcen in the North-West of Algeria, no epidemiological data on T1D in children are available.

The objective of this study was to assess the incidence of T1D in children under 15 years from the region of Tlemcen, in northwestern Algeria between January 1, 2015 and December 31, 2018.

## Methods

### Study Design and Data Collection

This retrospective study was conducted in Tlemcen, one of the largest cities in northwestern Algeria. This region, bordering on Morocco, is defined by a diverse geography, a Mediterranean climate and an Arab Muslim sociodemographic structure. According to the 2014 census, the population numbers 1,032,065 inhabitants and the population of children under 15 years was estimated at 267,597 children (male: 136,084; female: 131,513), accounting for 25.93% of the total population. There are five pediatric units in Tlemcen: pediatric department of the Mother and Child Specialized Hospital at Tlemcen’s Teaching Hospital, and pediatric departments of four Public Hospitals (PH), PH of Maghnia, PH of Ghazaouet, PH of Remchi and PH of Sebdou.

The mid-year estimates of the children population under 15 years were obtained from the annual statistical census data of the office of the Ministry of the Interior and from the regional Statistical Office of Tlemcen.

The diagnosis of T1D was made by the pediatric physician, according to the accepted criteria of the American Diabetes Association (16). The date of diagnosis of diabetes was accepted as the day of the first insulin injection. The months of diagnosis of T1D were sorted by seasons to examine the possibility of seasonality in the onset of childhood diabetes. Diabetic ketoacidosis at the time of diagnosis was observed and recorded as defined by the ISPAD Clinical Practice Consensus  Guidelines (17).

All children under 15 years of age living in the region of Tlemcen for at least six months prior to diagnosis, and presenting as newly diagnosed T1D for the period from from January 1, 2015 to December 31, 2018, were included. We excluded children with another type of diabetes (type 2 diabetes mellitus, neonatal diabetes, maturity onset diabetes of the young, and diabetes caused by other conditions).

The main source data on children diagnosed with T1D were based on the registers and the hospital records of the pediatric department of the Mother and Child Specialized Hospital at Tlemcen’s Teaching Hospital, and derived from the hospital records of pediatric departments of the four PH of Tlemcen. In the region of Tlemcen, all children under the age of 15 years, newly diagnosed with T1D are referred to these five pediatric units, as they are the only clinical institutions authorized to write a report for the initiation of insulin treatment and for follow-up.

The secondary independent data source of ascertainment was based on the Algerian social security system (Algerian national Health Insurance, ANHA). In Algeria, every child with T1D receives free treatment and diabetes is one of the chronic conditions which benefits from full coverage by the Algerian State (ANHA).

To measure case ascertainment and confirm the completeness of the recording, the capture-recapture method was used (18). This method would be expected to identify all new cases of children with T1D by capturing them in the first source and recapturing them in the second source in order to minimize the probability of underestimating the real number of new cases and to adjust accordingly the incidence of childhood T1D in the region.

The authors believe that a full census of all children under 15 years, newly diagnosed with T1D during the study period in the region of Tlemcen, was performed for this study.

This study was approved by the University Ethics and Deontology Council of the University of Tlemcen, Tlemcen, Algeria (approval number: CEDUT/DZ/019/117). Informed consent was obtained from the parents of children.

### Statistical Analysis

The average annual incidence rates were calculated by dividing the newly diagnosed cases of T1D in children aged under 15 years in a specific year, by population at risk aged under 15 years residing in Tlemcen in that year, and is expressed per 100,000 persons per year. Total average incidences were calculated by sex, by three pediatric age groups (0-4, 5-9 and 10-14 years) and by the season of the year at diagnosis.

The 95% confidence intervals (CI) of the annual incidence rates were calculated based on Poisson distribution. Independent chi-squared test was used to compare the rates between years, sexes and age groups, a p value (p) <0.05 was considered significant. Poisson regressions were used to analyze the changes in diabetes incidences with age, sex, season at diagnosis and year period. Statistical analysis was performed using the software R [R Core Team (2020). R: A language and environment for statistical computing. R Foundation for Statistical Computing, Vienna, Austria] (x64 3.3.2).

## Results

Overall ascertainment with capture-recapture method using the two independent sources was estimated to be 96% complete for the study period.

**Figure 1:**
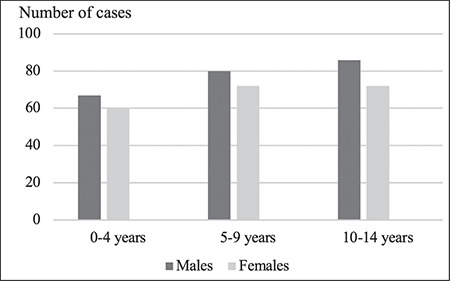
Number of new cases (males and females) of type 1 diabetes by age groups in the 2015-2018 period

During the period from January 1, 2015 to December 31, 2018, 437 new cases of T1D in children under 15 were registered in the region of Tlemcen consisting of 233 (53.32%) boys and 204 (46.68%) girls, with a male/female sex ratio of 1.14. Children were classified into three age groups: 29.06% of children diagnosed were under the age of five years, 34.78% aged between 5-9 years, and 36.16% of children aged 10-14 years (Figure 1).

The overall mean age at onset of T1D in this population was 7.51±4.12 years (95% CI: 6.56-8.35), with no significant difference between boys 7.46±4.14 years (95% CI: 6.40-8.62) and girls 7.56±4.11 years (95% CI: 6.70-8.21) (p>0.05).

The average annual incidence rate of T1D among children in these four years was 38.5 new cases per 100,000 persons under 15 years old (95% CI: 35.20-41.79) (boys: 40.51, 95% CI: 38.16- 42.85, girls: 36.49, 95% CI: 34.17-38.80). The difference in the incidence rate between boys and girls was only statistically significantly different in 2015 (p=0.00064), while for the other years of the study, there was no significant preferential difference between boys and girls (p>0.05). The incidence rates in 2015, 2016, 2017 and 2018 were respectively 36.6 (95% CI: 33.72-39.48), 38.7 (95% CI: 35.43-41.97), 39.3 (95% CI: 35.97-42.62) and 39.5 (95% CI: 36.12-42.87) per 100,000 respectively without significant difference between these four years of study. The number of cases and annual incidence rates by sex are presented in Table 1.

The incidence of T1D was lower in children of 0-4 years old years (31.11 per 100,000, 95% CI: 29.12-33.09) and higher in the 5-9 and 10-14 years age groups, with a peak of 44.78 per 100,000, 95% CI: 42.96-46.59) between 5-9 years, these differences between age groups of onset of childhood diabetes were not statistically significant (p>0.05). The annual incidence rates by sex and by age groups are presented in Table 2 and Table 3.

Poisson regression results show that the 5-9 years old group had 1.43 times risk, and the 10-14 years old group had 1.31 times risk compared to the 0-4 years old group (p<0.05).

The study of seasonality in the diagnosis of T1D showed that most cases in the region of Tlemcen were diagnosed in autumn (25.06%) and winter (28.25%), the cooler and rainier seasons of the year but fewer in spring and summer (24.45% and 22.24%, respectively), the warmer seasons of the year, but the seasonal variation were not statistically significant (p>0.05). This trend in onset seasonality was present in both sexes and in the three age classes. November was the month with the highest number of newly diagnosed children (9.83%) and June was the month with the lowest number of new cases (5.72%).

A total of 138 children (29.2%) had ketoacidosis at diagnosis. Diabetic ketoacidosis (DKA) was more common (53.62%, 74/138) in girls, but no significant difference between the two sexes. Regarding the frequency of DKA by age group, the difference between the frequencies of the different age groups was not statistically significant.

## Discussion

This study is the first to produce a reliable estimate of the incidence of T1D in children under 15 years old in Tlemcen. During the study of 2015 to 2018 inclusive, the incidence of T1D in children was estimated at 38.5 per 100,000 children under 15 years per year. Our results show that the region of Tlemcen presents a very high risk of T1D in children under 15 years.

In Algeria there are only a few reports of the epidemiological profile of T1D in children aged under 15 years. Nevertheless, the incidence in our childhood population is comparable to that of other studies conducted in Algeria. In 2016, in the capital Algiers, the incidence of T1D was 29.35 per 100,000 children under 15 years (19). In the region of Oran, the incidence of T1D in children under 15 was 31.12 per 100,000 in the period 2013-2017 (20). All these studies from Algeria report a T1D incidence in children in the “extremely high category” (incidence rate >20 per 100,000 persons per year) of the WHO DiaMond project classification for diabetes (21).

There is a clear difference in the incidence of childhood T1D in these different regions of Algeria. Similar differences in the incidences of T1D in children between regions of the same country are well documented (21,22,23).

During this study period, the incidence of childhood T1D ranged from 36.60 per 100,000 in 2015 to 39.50 per 100,000 in 2018, but due to the short period of our study, we cannot reliably estimate the rate of increase in the incidence of T1D in children under 15 years old in the region.

Worldwide, after the Nordic countries (Finland, Sweden, and Norway), some countries with an Arab population (Kuwait and Saudi Arabia) have the highest rates of T1D (9). In Africa, epidemiological data are incomplete and many countries have no studies on the incidence of T1D in children. The incidence of childhood diabetes in Tlemcen is clearly higher than in neighboring North African countries, notably Tunisia (7.7 per 100,000) during 1990-1999 (3), Libya (7.8 per 100,000) during 1991-2000 (24), Sudan (10.1 per 100,000) in 1990 (25), and Egypt (3.1 per 100,000) in 2011 (26). It is also higher than in some other MENA countries, notably Qatar (31.83 per 100,000) in 2016 (27), Iraq (8.0 per 100,000) in 2016 (28) and in Turkey (10.8 per 100,000) during 2011-2013 (23), but it was lower than that of eastern Saudi Arabia (52.93 per 100,000) in 2007 (29) and than that of Kuwait (40.9 per 100,000) during 2011-2013 (30). It should be noted that the studies carried out in most of the North African countries mentioned are relatively old. The high rate of incidence of T1D in our population in Tlemcen, compared to neighboring countries, is presumably due to as yet undetermined genetic or environmental factors, although the period between the older North African studies and the present study will account for some of the difference in incidence as there is a general global increase of 3% per annum.

In comparison with the Mediterranean countries, the incidence of diabetes in our pediatric population is higher that of Spain (22.84 per 100,000) during 2013-2016 (31), France (19.1 per 100,000) in 2015 (32), Italy (25.2 per 100,000) during 2009-2013 (33), Montenegro (18.8 per 100,000) in 2011 (34), Croatia (17.23 par 100,000) during 2004-2012 (35), and Cyprus 11.4/100,000 during 2011-2016 (36) but it remains lower than that Sardinia (51.0 per 100,000) during 2007-2009 (37).

In our study, the mean age of diagnosis of type diabetes was 7.51, which was high compared to Saudi Arabia (7.0 years) (38), but lower than in Kuwait, Spain and Turkey (respectively 8.1, 8.3, and 9.1 years) (30,31,39). It is notable that in this study, approximately one-third of children were diagnosed below 5 years of age, which would lower the average age of diagnosis of T1D in our pediatric population. Recent data from several regions of the world have also shown a large increase in the incidence of T1D in the youngest age-group (0-4) years (3,40). The incidence of childhood diabetes differs by age groups and is often reported to peak during the pubertal period. Moreover, an increase in the incidence of diabetes with age to puberty has been reported in several regions in the world (3). In this period of study, the highest incidence rates in Tlemcen were observed in the age groups of 5-9 years and 10-14 years. In this population, the incidence increases with age and peaks between 5-9 years, which is similar to studies conducted in Kuwait (30), in Italy (33), and in Finland (41). While Saudi Arabia (38), Turkey (23), Spain (31), Croatia (35) and some regions of Algeria (12,14,15), have described peak incidence of T1D in the 10-14 age group.

The incidence of childhood diabetes may differ by gender. In our population in Tlemcen, the number of incident cases of T1D is slightly higher in boys than in girls, but the incidence rates were only statistically different in 2015 between boys and girls. However, no significant difference in the incidence of childhood diabetes between boys and girls was observed in Algiers (19) and in several countries of the world (30,31,33,41). In contrast, a female predominance is observed in Saudi Arabia (38), and a male excess has been observed in Hungary (42) and in Finland (43).

The seasonality of the onset of childhood diabetes has been confirmed by the Eurodiab study, and the existence of a winter peak in the onset of childhood diabetes has been described in different regions of Europe (44). During this 4-year period, we noted a predominance of winter peak without significant seasonal variation in the onset of T1D in the region of Tlemcen. Similar findings were reported in other regions of Algeria (12,20) and in other countries (26,27), where more cases of childhood T1D occurs in the winter season. In contrast, higher incidences were observed in the spring season in Diyarbakir in the Southeast region of Turkey (39). This seasonal variation is supportive of the hypothesis of a viral trigger for childhood diabetes (8), principally the hypothesis of the triggering infection being due to enterovirus (45).

Worldwide, the DKA frequency at diagnosis of T1D varies from 12.8% to 80% (46). However, the frequency of DKA at diagnosis of T1D in our study was 29.2%. Recently in 2016 in the capital Algiers, 17.6% of children aged 0-14 years had DKA (19). Compared to previous studies from other countries, the frequency of DKA in our diabetic children (29.2%) was higher than in Spain (17.8%) (47) and in France (14.8%) (48), but was lower than that reported in Kuwait (33.6%) (49), in Saudi Arabia (40%) (29) and in Turkey (65.9%) (39).

### Study Limitations

However, this study presented some limitations. First, it was possible that some cases of monogenic diabetes in children was misclassified because genetic testing for monogenic causes were not routinely practiced in all new children diagnosed with diabetes before nine months of age. Second, due to the short duration of the study, we cannot reliably describe trends of childhood diabetes in this region. Third, we cannot explain the causes of the very high incidence of childhood diabetes in our population because the data on genetic susceptibility factors and environmental triggers are limited.

## Conclusion

The incidence of childhood T1D in Tlemcen in Northwest Algeria was 38.5 per 100,000. This incidence is in the “extremely high” category of the WHO project classification for diabetes giving the region a very high risk. Other large-scale epidemiological studies at the national level should be conducted to determine the incidence of childhood diabetes mellitus in Algeria. In addition, further studies on genetic and environmental risk factors for T1D are needed to better explain the high incidence of T1D in children in Algeria.

## Figures and Tables

**Table 1:**
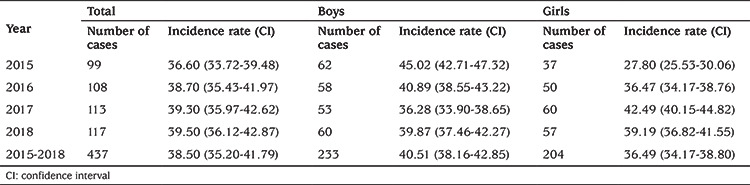
Number of cases and annual incidence of type 1 diabetes among children under 15 years age per 100,000 persons per year (95% confidence interval) by sex in Tlemcen between 2015 and 2018

**Table 2:**
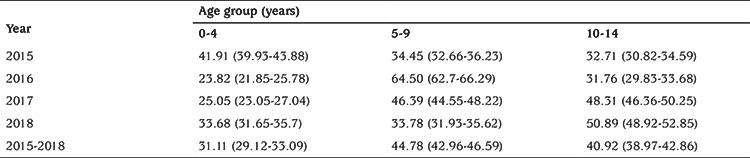
Annual incidence of type 1 diabetes among children under 15 years age per 100,000 persons per year (95% confidence interval) by age groups in Tlemcen between 2015 and 2018

**Table 3:**
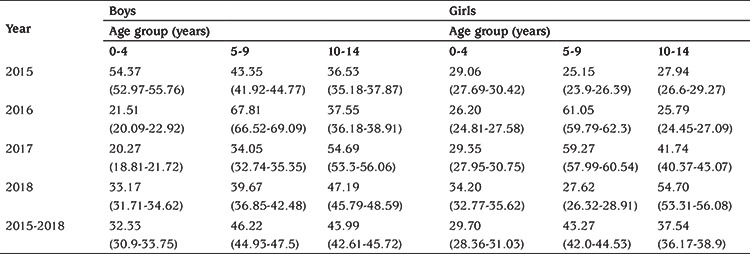
Annual incidence of type 1 diabetes among children under 15 years age per 100,000 persons per year (95% confidence interval) by sex and by age groups in Tlemcen between 2015 and 2018
